# Effect of unilateral adrenalectomy on the quality of life of patients with lateralized primary aldosteronism

**DOI:** 10.1186/s12893-018-0432-1

**Published:** 2019-04-24

**Authors:** Marilisa Citton, Giovanni Viel, Francesca Torresan, Gian Paolo Rossi, Maurizio Iacobone

**Affiliations:** 1 Endocrine Surgery, Department of Surgery, Oncology and Gastroenterology, University of Padua, Via Giustiniani 2, 35128 Padova, Italy; 2 Hypertension Clinic, Department of Medicine, University of Padua, Via Giustiniani 2, 35128 Padova, Italy

**Keywords:** Primary aldosteronism, Quality of life, Depression status, Adrenalectomy

## Abstract

**Background:**

Primary aldosteronism (PA) is associated with an increased prevalence of anxiety and depression. Subnormal quality of life (QoL) scores in PA patients may be improved after surgical treatment. The aim of the study was to assess the impact of surgery on health-related QoL and depression status of patients suffering from PA, comparing the results with a control group of patients undergoing surgery for non-secreting adrenal tumors.

**Methods:**

Data on QoL and depression status were prospectively collected, from January 2014 to January 2017, before, early after surgery (at 1 month) and at late follow up (at least 6 months) in patients with unilateral PA and in a control group with non-secreting adrenal tumors submitted to unilateral laparoscopic adrenalectomy. QoL was assessed using the Short Form 36 (SF-36) Health Survey for Physical (PCS) and Mental Component (MCS); the depression status by a 20-item depression scale (DS) questionnaire.

**Results:**

Twenty-six PA patients and 15 controls were recruited. Biochemical cure of the disease was achieved following surgery in all PA patients; hypertension was cured in 31% of cases and improved in the remaining 69% of cases. No morbidity occurred in both groups. There were no significant differences between PA patients and controls concerning demographics, preoperative PCS, MCS and DS values. In patients with PA, MCS values improved at early (42.72 ± 13.68 vs 51.56 ± 9.03, *p* = 0.0005) and late follow up (42.72 ± 13.68 vs 51.81 ± 7.04, *p* < 0.0001); also DS values improved at early (15.92 ± 11.98 vs 8.3 ± 8.8, *p* = 0.0002) and late follow up (15.92 ± 11.98 vs 4.57 ± 6.11, *p* < 0.0001). In PA patients PCS values significantly improved at late follow up (51.02 ± 8.04 vs 55.85 ± 5.1, *p* = 0.013). Also in controls an improvement of MCS and DS scores was found at early and late follow up compared to preoperative values, while no significant differences in PCS were found.

**Conclusions:**

Both PA and non-secreting adrenal tumors affect health-related QoL, worsening MCS and DS scores. Adrenalectomy is effective in curing PA, and improving MCS and DS scores at early and late follow-up, in patients with PA and non-secreting adrenal tumors. In PA patient surgery also significantly improves PCS at late follow up.

## Background

Primary aldosteronism (PA) due to unilateral or bilateral overproduction of aldosterone, is the most common cause of endocrine hypertension, and it has been reported in more than 11% of referred hypertensive patients [1, 2].

The lateralization of aldosterone hypersecretion is crucial, because only patients with unilateral PA are held to be surgically curable [3]. Unilateral laparoscopic total adrenalectomy is currently the preferred strategy in patients with lateralized excess [4–7].

Besides leading to increased cardiovascular morbidity and mortality [1], PA has been claimed to be associated to higher prevalence of anxiety and depression, with potential impact on health-related quality of life (QoL), through mechanisms and pathways that remain to be clarified [8, 9].

Undoubtedly, both surgical and medical treatment of PA can control hypertension and severe cardiovascular damages in the long term [3]. However, whether these two strategies have the same beneficial effects on QoL and depression remains uncertain. In fact, they show substantial differences, as surgery eliminates the source of aldosterone excess, while mineralocorticoid receptor antagonists simply control it [10, 11].

Measurement of QoL investigates the functional status of the individual and the patient’s appraisal of health, allowing assessment of the impact of a disease and/or treatment from the patient’s perspective.

Therefore, only few studies investigated health-related QoL has been in patients suffering from PA [12, 13], reporting subnormal scores compared to normal population. A recent systematic review, not focusing on this issue, [14] highlighted that, in patients with PA, health-related QoL as well as scores for depression and anxiety [11] ameliorated after surgery with respect to medical treatment.

However, it could not be excluded that non-specific psychological effects secondary to surgery could be at least partly responsible for the improvement in QoL [10].

The aim of the present study was, therefore, to assess the impact of surgery on health related QoL (both in Mental and Physical components) and depression status of patients suffering from PA and to compare them with a control group of patients with non-secreting adrenal tumor who also underwent adrenalectomy.

## Methods

Data were prospectively collected from January 2014 to January 2017 at Endocrine Surgery Unit of Padua University Hospital, Italy.

The present prospective non-randomized study included patients with unilateral PA and a control group of patients with non-secreting adrenal tumor submitted to laparoscopic transperitoneal adrenalectomy by flank approach performed by the same surgeon (M.I.). The institutional ethics committee approved the study and informed consent was obtained from all patients.

PA was diagnosed based on a plasma aldosterone concentration greater than 15 ng/dL and an aldosterone/renin ratio greater than 40 ng/dL:ng/mL/h, measured after washout of interfering drugs or after changes of the drug treatment as previously detailed [15]. The diagnosis was confirmed by saline infusion and/or the captopril test [15].

Arterial hypertension was defined by a systolic blood pressure (BP) of 140 mmHg or greater, diastolic BP of 90 mmHg or greater, or both and/or the presence of antihypertensive medical treatment.

Surgery was performed in patients with lateralized PA, according to the results of preoperative lateralizing techniques. Lateralizing techniques included adrenal venous sampling (AVS), CT scan and/or MR as previously described [16].

AVS was performed with bilateral simultaneous catheterization, by using one catheter for each adrenal vein. Successful selective catheterization was usually confirmed when the ratio between cortisol concentration in each adrenal vein and the inferior vena cava was greater than 1.1; unilateral aldosterone hypersecretion was usually confirmed when the ratio of adrenal vein aldosterone concentration to the homolateral cortisol concentration on the side with the higher ratio over the contralateral aldosterone to cortisol ratio (AVS ratio) was greater than 2.

The control group was composed by patients suffering from non-secreting adrenal tumor, defined as asymptomatic adrenal mass, incidentally detected on imaging not performed for suspected adrenal disease, in patients without glucocorticoid, catecholamine or mineralcorticoid hypersecretion (assessed by plasmatic ACTH, 24-h urinary free cortisol levels and 1 mg overnight dexamethasone suppression test, 24-h urinary catecholamine or metanephrine levels and plasma renin and aldosterone levels, respectively). In these patients, surgery was indicated by the presence of adrenal mass with suspicious radiological findings (even without evidence of local invasion or distant metastases), evidence of significant tumor growth during follow-up imaging and/or patient preference. Patients with adrenal or extra-adrenal malignancies or any psychiatric disorders were not included in the study.

The surgical procedure was performed with the patient in lateral decubitus flank position. Pneumoperitoneum (at 12–14 mmHg by CO_2_) was made by Hasson cannula inserted by open technique. A subcostal port was placed for the laparoscope, and two/three other 5/10 mm ports sited. For right adrenalectomy the liver was mobilized and retracted and the right medium adrenal vein was identified by following the lateral edge of the vena cava. The main right adrenal vein was identified and divided early. Then the adrenal branches from the inferior phrenic artery, aorta, and the renal artery were divided. On the left side the colonic flexure was mobilized and the splenorenal ligament dissected, allowing the fall of the spleen medially and the identification of the tail of the pancreas. The avascular plane separating adrenal gland from the tail of the pancreas was opened, allowing the view of the inferior adrenal vein, going into the left renal vein. The inferior adrenal vein was divided and then the gland was dissected and removed. Small arterial vessel from superior, medium and inferior pedicle were coagulated. In all cases, the adrenal gland was removed via a retrieval bag [7, 17].

Health-related QoL and depression status, were assessed preoperatively (at the time of hospital admission) and postoperatively at 1-month outpatient control and at long term (at least 6 months after surgery).

QoL was evaluated using the Italian version of Short Form 36 (SF-36) Health Survey for a Physical (PCS) and a Mental Component (MCS). The SF-36 is a 36 item self-administered questionnaire measuring QoL across eight domains obtaining eight scaled scores, which are the weighted sums of the questions in their section. These domains are physical functioning, social functioning, role limitations due to physical problems, role limitations due to emotional problems, vitality, bodily pain, general health perceptions, and general mental health. PCS and MCS are a summary of physical and emotional QoL respectively. Scores may range from 0 (poorest health status) to 100 (best health status). The depression status was evaluated using a 20-item depression scale (DS) questionnaire; the score may range from 0 (best status) to 60 (poorest status) [18, 19]. The results were compared with published normative values for the Italian population [20].

Records of the patients were reviewed to gather relevant demographics, body mass index (BMI, defined as body weight (kg)/height (m^2^), normal values 20 to 24.9), hormonal parameters (including glucocorticoid, mineralcorticoid and catecolamine assays), BP values, number of antihypertensive drugs, side and size of adrenal masses, intra and postoperative morbidity and definitive pathology.

Postoperative follow-up data (including hormonal, BP parameters, number of antihypertensive drugs), were assessed 1 month after surgery and at long-term.

Results were expressed as absolute numbers, ratio, percentage, mean (± standard deviation) or median (range).

Statistical analysis was performed using Fisher’s exact test for categorical variables, Student’s paired *t* test, Wilcoxon matched-paired test, Mann–Whitney *U* test, as appropriate. *p* < 0.05 was considered statistically significant.

## Results

Twenty-six PA patients and 15 patients with non-secreting adrenal tumor undergoing laparoscopic transperitoneal adrenalectomy were recruited.

No significant differences were found between PA patients and controls concerning demographics, BMI, side and dimension at preoperative imaging of the mass (Table 1). The size of the adrenal mass at preoperative imaging was significantly higher in controls than in PA patients (*p* < 0.0001) (Table 1).

**Table 1:** Demographics, and general features in (PA patients) patients with Primary Aldosteronism and controls undergoing laparoscopic adrenalectomy

	PA Patients (*n* = 26)	Controls (*n* = 15)	*P* value
Sex (female/male)	10/16	6/9	0.92
Age (years)	54 ± 11	56 ± 9	0.54
Body Mass Index (kg/m^2^)	26.6 ± 4	27.4 ± 6	0.95
Side of the mass (left/right)	12/14	9/6	0.59
Size of the mass (mm) median (range)	15 (6–30)	44 (25–60)	< 0.0001

*PA* Primary aldosteronism

Hypertension was present in all patients with PA and in five patients with non-secreting adrenal tumor (*p* < 0.0001); hence, the mean systolic and diastolic BP were significantly higher in PA patients than in controls (154 ± 19 vs 125 ± 9 mmHg, *p* < 0.0001 and 91 ± 12 vs 75 ± 8 mmHg, *p* = 0.0002, respectively); likewise, the number of antihypertensive drugs was significantly higher in PA patients than in controls (3.42 ± 1.47 vs 0.60 ± 0.99, *p* < 0.0001).

Preoperative MCS and DS scores were impaired in PA patients compared to normal Italian reference population; similar findings were found also in the control patients.

No significant differences were found between PA patients and controls concerning preoperative PCS (51.02 ± 8.04 vs 51.16 ± 9.63, *p* = 0.75), MCS (42.72 ± 13.68 vs 39.39 ± 12.81, *p* = 0.39), and DS values (15.92 ± 11.98 vs 16.26 ± 12.56, *p* = 0.91).

All patients underwent uneventful laparoscopic surgery; no conversion to open approach was performed, no blood transfusion was required and no intra- or post-operative morbidity occurred in both groups.

Pathological specimens revealed benign adrenal tumors in all cases in both groups.

### Early follow-up

At one month postoperative follow up, PA was biochemically cured in all patients, according to the normalization of the aldosterone/renin ratio and serum potassium levels; hypertension was cured in 10 cases (38%), and improved in the remaining 16 cases (62%). The systolic and diastolic BP were significantly reduced (from 154 ± 19 to 130 ± 15 mmHg, *p* < 0.0001 and from 91 ± 12 to 78 ± 9 mmHg, *p* < 0.0001); also the mean number of antihypertensive drugs was significantly reduced (from 3.42 ± 1.47 to 1.15 ± 1.1, *p* < 0.0001).

**Fig. 1:**
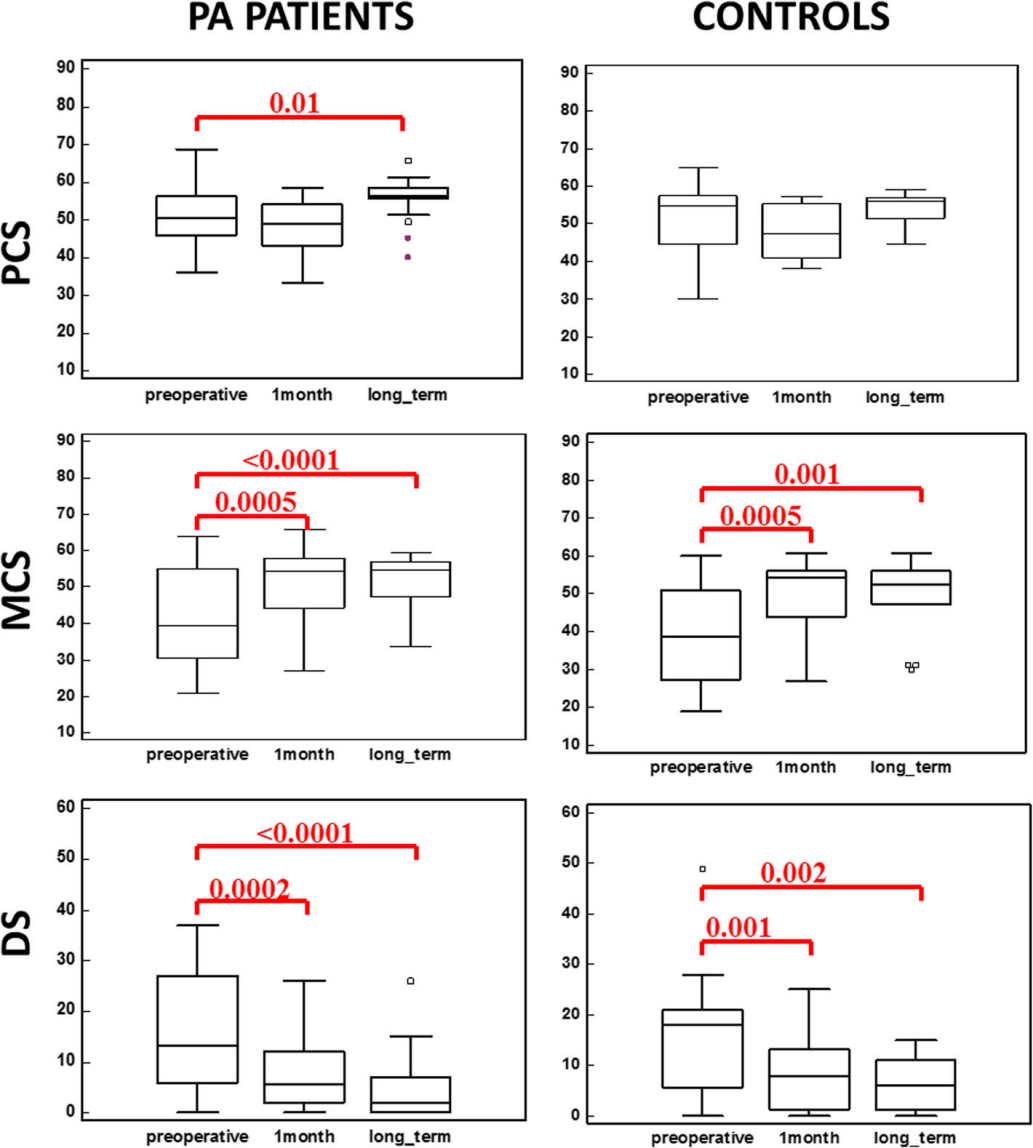
PCS, MCS and DS values in PA patients and controls, preoperatively, 1 month after surgery and at long term. *PA: primary aldosteronism; PCS: Physical Component Score; MCS: Mental Component Score; DS: depression scale*

In patients with PA, MCS values significantly improved (42.72 ± 13.68 vs 51.56 ± 9.03, *p* = 0.0005) (Fig. 1), mainly due to an amelioration in the “mental health” (42.7 ± 15.7 vs 53.17 ± 7.33, *p* = 0.001) and “emotional role” (45.5 ± 11.83 vs 51.23 ± 9.84, p = 0.001) scores. Also DS values significantly improved (15.92 ± 11.98 vs 8.3 ± 8.8, *p* = 0.0002). Conversely, no quite significant differences were found regarding PCS scores (51.02 ± 8.04 vs 48.01 ± 6.85, *p* = 0.07) (Fig. 1).

In controls, no significant changes in BP levels and number of antihypertensive drugs was found; also in the five hypertensive patients no changes in BP values and number of antihypertensive drugs were detected after adrenalectomy.

Also in controls MCS (39.39 ± 12.81 vs 50.62 ± 8.68, *p* = 0.0005) and DS (16.26 ± 12.56 vs 7.86 ± 7.19, *p* = 0.001) values improved after surgery, mainly due to a significant amelioration in the “mental health” (46.69 ± 12.01 vs 53.79 ± 6.36, *p* = 0.006) and “emotional role” (33.19 ± 13.69 vs 46.93 ± 9.26, p = 0.001) scores (Fig. 1). No significant differences were found regarding PCS values (51.16 ± 9.63 vs 48.60 ± 6.99 *p* = 0.26).

No significant differences were found between PA and control patients concerning postoperative MCS and PCS and DS (*p* = 0.69, *p* = 0.81 and *p* = 0.93, respectively).

### Long term follow up

At long term follow up (median 8 months, range 6–13), all patients that underwent surgery for PA were still biochemically cured, but two patients that at one month had achieved the cure of hypertension, restarted antihypertensive therapy; thus, the hypertension cure was achieved in 8 patients (31%).

PA patients had systolic and diastolic BP values significantly reduced (from 154 ± 19 to 129 ± 13 mmHg, *p* < 0.0001 and from 91 ± 12 to 79 ± 8 mmHg, *p* < 0.0001), compared with preoperative values; also the mean number of antihypertensive drugs was significantly reduced (from 3.42 ± 1.47 to 1.28 ± 1.2, *p* < 0.0001).

These patients had no significant differences in BP values and number of antihypertensive drugs one month after surgery and at long term follow up.

In patients with PA, a significant improvement in PCS (51.02 ± 8.04 vs 55.85 ± 5.1, *p* = 0.013), MCS (42.72 ± 13.68 vs 51.81 ± 7.04, *p* < 0.0001) and DS (15.92 ± 11.98 vs 4.57 ± 6.11, *p* < 0.0001) values was described, compared with preoperative period; PCS values improved significantly, compared with values recorded one month after surgery (*p* = 0.0002) (Fig. 1).

At late follow up, also in controls MCS (39.39 ± 12.81 vs 49.30 ± 10.46, *p* = 0.001) and DS values (16.26 ± 12.56 vs 6.80 ± 5.64, *p* = 0.002) improved after surgery, mainly due to a significant amelioration in the “mental health” (46.69 ± 12.01 vs 54.64 ± 7.87, *p* = 0.007) and “emotional role” (33.19 ± 13.69 vs 51.62 ± 9.87, *p* < 0.0001) scores (Fig. 1). No significant differences were found regarding PCS values (51.16 ± 9.63 vs 53.91 ± 4.30 *p* = 0.25).

No significant differences were found between PA and control patients concerning MCS and PCS and DS (*p* = 0.58, *p* = 0.16 and *p* = 0.18, respectively).

## Discussion

PA is a common, albeit often overlooked, cause of hypertension which is often severe and/or drug resistant and, therefore, associated with cardiovascular damage and a worse prognosis [21, 22]. Moreover, it implies clear cut alterations of the renin-angiotensin-aldosterone system, which suggests that it entails multiple reasons to imply a worsened QoL, given that the derangements of this important system can affect QoL [8, 9].

Hypertension is an important factor for reduced QoL: an impairment of QoL has been reported in patients with essential hypertension compared to normal controls in the somatization and psychological distress [23].

Along with this hypothesis, at the best of our knowledge, Health-related QoL in patients suffering from PA has been previously investigated only by 3 studies [12, 13, 24].

In 2010, Sukor et al. [24] examined health-related QoL in 22 patients with unilateral PA before and after unilateral adrenalectomy at 3 and 6 months, using SF-36 questionnaire. They found a significant improvement in the QoL of these patients both in physical and mental condition. However, they did not examine depression and anxiety.

In 2011, the same authors [12] compared the results of the previous study with those from 21 patients with bilateral PA, before or after commencing medical treatment, and with those of the normal Australian population. They confirmed that PA patients had subnormal QoL scores compared to normal population. Moreover, they described that QoL improved in all patients with bilateral PA undergoing medical treatment, but more slowly and to a lesser degree than in patients undergoing surgery for unilateral PA.

In 2012, Kunzel et al. [13] published the results of a cross sectional study on the data from German Conn Registry examining health-related QoL using SF12 questionnaire, in which they investigated acute impairment of QoL and long-term treatment effects in patients with PA. The study included 132 patients with PA, stratified according to the treatment status: 27 newly diagnosed, untreated; 52 in chronic medical treatment, 49 patients treated with adrenalectomy. The study confirmed that PA patients had a worse physical and mental condition than the normal German reference population; untreated and medically treated patients reported the lowest scores.

Our study differed substantially from these previous studies, since we enrolled as controls patients undergoing the same surgical procedure for non-functioning adrenal mass.

This allowed adjustment for the potentially confounding effect of being harboring an adrenal tumor and of being submitted to adrenalectomy. Moreover, our study was aimed to clarify the impact of surgery on health-related QoL (both in Mental and Physical components) and for the first time on depression status, in patients suffering from PA.

In agreement with previous studies, we confirmed that patients with PA have an impaired QoL and depression status compared with normal population, as assessed by worse MCS and DS scores.

In PA patients, MCS (especially in the mental health and emotional role dimensions) and DS values significantly improved 1 month after surgery and at long term follow-up, confirming the beneficial effect of adrenalectomy previously reported [12]. However, we failed to find significant differences with the controls, since also these patients (without aldosterone excess) showed an impaired preoperative MCS and DS scores, and a significant amelioration one month and at long term after surgery. Interestingly, also in controls MCS improvement was mainly due to mental health and emotional role amelioration. However, the amelioration was more evident in PA patients.

Even if controls and PA patients were similar according to demographics and type of surgical procedure, the former had lower BP levels, larger adrenal masses and underwent surgery mainly because of a suspicion of malignancy, while there was not suspicion for the latter.

Thus, it remains unclear if it may be related to the reduction of BP levels or antihypertensive drug treatment (with a possible reduction of drug-related size effects), to the aldosterone/renin system normalization or a non-specific psychological effect of surgery.

We may argue that in control population the thought of impending surgery and the uncertain nature of the non-secreting adrenal mass might have affected the mental component and increased the depression status before surgery. Obviously, both factors might have been solved after surgery, since in all cases definitive pathology described a benign adrenal tumor; however, other psychologic effects of adrenalectomy may not be excluded, as previously reported [10].

In the present study, no significant amelioration of PCS scores was detected in PA patients one month after surgery; this finding might be related to the sequelae of recent surgery. In fact, in PA patients the improvement in PCS values become evident at long term follow-up. This is in agreement with previous studies that demonstrated at 3 months a significant increase of physical condition, after disease cure and BP normalization or amelioration [24].

In the control group, no significant variations were detected in PCS values at one month and at long term, compared with preoperative values.

However, some limitations to the present pilot study that might have biased the results should be underlined, including the limited number of cases and length of follow up. Moreover, the mental component of health–related QoL is difficult to explore; the SF-36 and DS questionnaires are not disease specific. Furthermore, the administration of the baseline questionnaire during hospitalization for surgery, when factors such as optimism or anxiety and fear of surgery could have some effects, might have affected the results in terms of worsening QoL and depression as compared to the normal population. However, if any, the effect was likely similar in both PA and controls.

## Conclusions

PA affects the health-related QoL, worsening the mental component and the depression status. Adrenalectomy is effective in curing PA, and improves the mental component of health-related QoL and depression status at 1 month and at long term. At long term, surgery determines an improvement also in the physical component of health-related QoL of PA patients.

The role of hormonal cure of PA and the possible weight of the psychological effects of surgery itself in affecting QoL need to be further explored, since some relevant results may be observed also in patients undergoing surgery for non-secreting adrenal masses. Further studies are needed to confirm these results at a longer follow up and with a larger population.

## Abbreviations

ACTH: Adrenocorticotropic hormone
AVS: Adrenal vein sampling
BMI: Body mass index
BP: Blood pressure
CT scan: Computed tomography
DS: Depression scale questionnaire
MCS: Health Survey for Mental Component
MR: Magnetic resonance
PA: Primary aldosteronism
PCS: Health Survey for Physical Component
QoL: Quality of life
SF-36: Short Form 36
